# Detection of *Staphylococcus epidermidis* by a Quartz Crystal Microbalance Nucleic Acid Biosensor Array Using Au Nanoparticle Signal Amplification

**DOI:** 10.3390/s8106453

**Published:** 2008-10-21

**Authors:** Han Xia, Feng Wang, Qing Huang, Junfu Huang, Ming Chen, Jue Wang, Chunyan Yao, Qinghai Chen, Guoru Cai, Weiling Fu

**Affiliations:** 1 Department of Laboratory Medicine, Southwest Hospital, Third Military Medical University, Chongqing 400038, P.R China. E-Mails: hxia16@gmail.com; wangfnew@gmail.com; dr.q.huang@gmail.com; junfu_huang@yahoo.com; chenming1971@yahoo.com; xiaojue21@gmail.com; yao_yao24@yahoo.com; cqh1969@yahoo.com.cn.; 2 The 26th Research Institute, Chinese Electronic Scientific and Technical Group Company, Chongqing 400060, P.R China. E-Mail: guorucai18@yahoo.com.cn.

**Keywords:** Au nanoparticle, biosensor, *S. epidermidis*, quartz crystal microbalance

## Abstract

*Staphylococcus epidermidis* is a critical pathogen of nosocomial blood infections, resulting in significant morbidity and mortality. A piezoelectric quartz crystal microbalance (QCM) nucleic acid biosensor array using Au nanoparticle signal amplification was developed to rapidly detect *S. epidermidis* in clinical samples. The synthesized thiolated probes specific targeting *S. epidermidis* 16S rRNA gene were immobilized on the surface of QCM nucleic acid biosensor arrays. Hybridization was induced by exposing the immobilized probes to the PCR amplified fragments of *S. epidermidis*, resulting in a mass change and a consequent frequency shift of the QCM biosensor. To further enhance frequency shift results from above described hybridizations, streptavidin coated Au nanoparticles were conjugated to the PCR amplified fragments. The results showed that the lowest detection limit of current QCM system was 1.3×10^3^ CFU/mL. A linear correlation was found when the concentration of *S. epidermidis* varied from 1.3×10^3^ to 1.3×10^7^ CFU/mL. In addition, 55 clinical samples were detected with both current QCM biosensor system and conventional clinical microbiological method, and the sensitivity and specificity of current QCM biosensor system were 97.14% and 100%, respectively. In conclusion, the current QCM system is a rapid, low-cost and sensitive method that can be used to identify infection of *S. epidermidis* in clinical samples.

## 1. Introduction

*Staphylococcus epidermidis*, a major skin flora component, was generally considered a contaminant with no or very low virulence when isolated from the blood of hospitalized patients. However, during the last two decades, changing trends in the practice and progress of medical devices in the supportive and critical care of hospitalized and chronically ill patients resulting in the emergence of *S. epidermidis* as a leading cause of nosocomial blood infections [1-3]. In most infections, *S. epidermidis* is always multi-resistant to antibiotics and forms biofilms, and the ability to escape from host immune defenses are regarded as the main virulence determinants of *S. epidermidis* [2, 4]. In the last decades, various techniques have been developed for the clinical diagnosis of *S. epidermidis* infections, such as conventional blood culture techniques, enzyme-linked immunosorbent assay, immunoassay, sequence analysis, and so on. These conventional methods are generally reliable but time-consuming, laborious and expensive. Therefore, it is desirable to explore some simple, sensitive, and low-cost diagnostic methods to detect *S. epidermidis* in clinical blood samples.

Because of sensitivity, simplicity and cost-effectiveness, piezoelectric quartz crystal microbalance (QCM) biosensors have showed attractive roles in various molecular analysis techniques [5-8]. The operating principle of QCM biosensor is based on mass-frequency shifts resulted from the interaction between the sensing biomolecules immobilized on its crystal surface and the targeted biomolecules. The formula of QCM mass-frequency effect was first reported by Sauerbrey [9] and later extensively developed by others [10, 11]:

(1) $$\Delta F=‐2.26\times 10^{6}F^{2}\Delta m/A$$

in which the ΔF is the measured frequency change (Hz) of the coated crystal, F is the fundamental resonance frequency (MHz) of the crystal, A is the area coated, and Δm is the mass deposited. A linear relationship exists between deposited mass and frequency response for quartz crystals. During the last decades, QCM has been extensively investigated as a transducer in hybridization based nucleic acid biosensors for the detection of gene mutation [12, 13], genetically modified organisms [14], and foodborne pathogens [15-17]. However, for most of the previously reported nucleic acid biosensors for bacterial detection, their sensitivity and detection limit are still difficult to meet the demand of clinical analysis.

In order to improve the detection sensitivity of QCM nucleic acid biosensors, various signal amplification strategies have been developed, such as anti-dsDNA antibodies [18], liposomes [19], enzymes [20], RecA protein [5] and nanoparticles [21-24]. Among these amplifiers, due to relatively larger mass compared with the targeted DNA, nanoparticles have promising applications to effectively improve the detection limit and sensitivity in the QCM DNA detection [21-25]. Two methods of nanoparticles signal amplification have been developed to extend the limits of DNA detection. One of the “nanoparticle amplifier” methods utilizes sandwich hybridization of specific probe functionalized nanoparticles, target DNA, and surface capture probes [21, 23]. The other method is to label the DNA targets with nanoparticles through ligands such as biotin on the targets [22, 24]. The latter one is simpler and especially suitable for the detection coupled with PCR because the DNA targets can be easily labeled with the ligands during the PCR reaction. Au nanoparticles have been demonstrated as a good nanomaterials to enhance the sensing performance of QCM biosensors due to their structural features and biocompatibility [26, 27], but Au nanoparticle signal amplification based QCM nucleic acid biosensors for the detection of *S. epidermidis* in clinical samples have not previously been reported.

In this study, a QCM nucleic acid biosensor array based on Au nanoparticle signal amplification was developed to rapidly detect *S. epidermidis* in clinical samples. The basic principle of current QCM biosensor system was as follows: after the biotinylated target DNA of *S. epidermidis* was captured through hybridization by the single-stranded DNA probes which were self-assembled on the QCM crystal surface, the hybridization signal was then amplified using the streptavidin-coated Au nanoparticles. The sensitivity and specificity of the QCM system were evaluated. The application of the QCM system was tested in real clinical blood samples.

## 2. Experimental Section

### 2.1. Reagents and oligonucleotides

The oligonucleotides (Table 1) were synthesized by Shanghai Bioengineering Company (Shanghai, P.R. China). The universal primers were used for amplification of the 16S rRNA gene from *S. epidermidis*. A 20-base oligonucleotide modified at 5′-end with C6-SH was used as an ssDNA probe. To optimize the performance of the nucleic acid biosensor array, the oligonucleotides that was complementary to the thiolated probe and labeled with biotin at 5′-end was used as positive control.

The biotinylated oligonucleotide with same sequence of probe has been used as negative control under matching working conditions. Streptavidin-coated Au nanoparticles (GoldMag™-AS Streptavidin) with 5 μm average diameter were purchased from Shanxi Lifegen Company (Xian, P.R. China).The binding capacity of streptavidin-coated Au nanoparticles to biotin was determined as 3000 pmol biotin/mg particles. Phosphate buffered saline (PBS), 30% hydrogen peroxide, 98% sulfuric acid, sodium chloride, hydrogen chloride, sodium hydroxide and 6-mercaptohexanol were purchased from Sigma Aldrich (St. Louis, MO, USA). The TIANamp Bacteria Kit (Tiangen, Beijing, P.R. China) was used to isolate bacterial genomic DNA. Other chemicals used were at analytical reagent grade, and super-pure water (18.2 MΩ) was used throughout.

### 2.2. Apparatus

**Figure 1.**
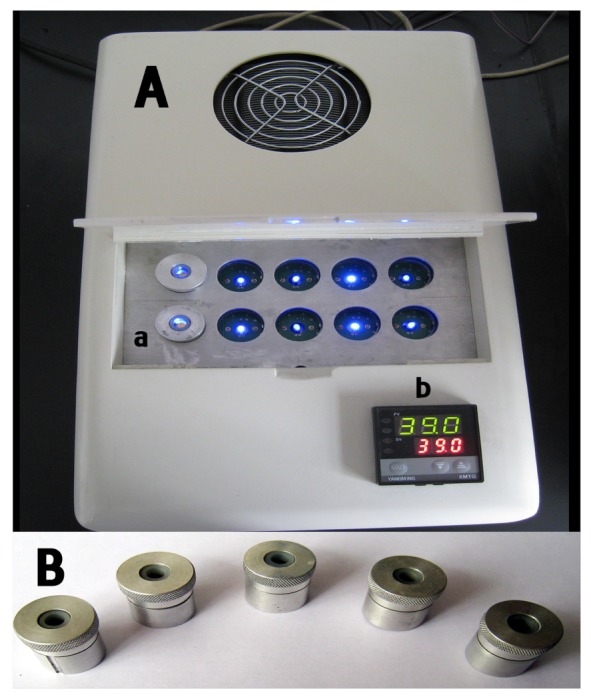
2×5 QCM nucleic acid biosensor array and detection wells. **(A)** Picture of QCM nucleic acid biosensor array detector. 2×5 detection wells and thermal controller system are indicated as follows: **(a)** 2×5 detection well array, **(b)** thermal controller system. **(B)** Picture of five detection wells.

AT-cut, 10 MHz quartz crystals (13 mm in diameter, 0.15 mm in thickness) with polished Au electrodes (4 mm in diameter) coated on both sides were obtained from the 26th Research Institute, Chinese Electronic Scientific and Technical Group Company (Chongqing, P.R. China). The QCM detector (Figure 1A), including electronic oscillation circuit, voltage stabilizer, thermal controller system, and 2×5 detection wells, was made by the Jialing Group of China. The experimental data were analyzed by a personal computer with software BIDE 2.11(self-developed) in real time, which was interfaced with the QCM detector. The detection cells were self-designed such that only one side of the quartz was exposed to the solution, and each of them was driven by an independent electronic oscillation circuit, without mutual interference. Temperature was controlled (± 1 °C) by an air thermostat and fluctuations gave negligible frequency variations.

### 2.3. Standard bacterial strains and clinical bacterial samples

Standard bacterial strains including *S. epidermidis* (ATCC 12228), *P. aeruginosa* (ATCC 27853), *S. aureus* (ATCC 25923), *E. coli* (ATCC 25922), and *K. pneumoniae* (ATCC 700603) were obtained from the American Type Culture Collection (ATCC; Manassas, VA, USA). Pure bacterial strain cultures were grown in brain heart infusion broth (Difco Laboratories, Detroit, MI, USA) at 37 °C for 20 h before the bacterial concentrations were determined by conventional spread-plating method using tryptic soy agar (TSA, Difco).

After automatic detection and analysis of BacT/Alert blood culture system (bioMérieux, Inc., Durham, NC, USA), blood samples that were positive infection of *S. epidermidis* were collected from 35 patients and the negative blood samples were collected from 20 healthy volunteers. All blood samples were collected from the Southwest Hospital (Chongqing, P.R. China) between July 2006 and August 2007. Written informed consent had been obtained from all patients or their family members. And the study was approved by the Ethics Boards of the Third Military Medical University. The BacT/Alert bottles were removed from the incubator, aliquots of 2-mL of the blood culture suspensions were taken aseptically with a needle syringe. Each aliquot was equally divided before detection, with one part for routine diagnostics, and the other part for DNA isolation. All blood cultures containing *S. epidermidis* used in the evaluation study were further confirmed by using conventional microbiological techniques.

### 2.4. DNA extraction and PCR amplification

The extraction of DNA from each concentration of standard bacterial strains was performed with a TIANamp Bacteria Kit followed by the manufacture's protocol. Total DNA from blood culture bottles was extracted and purified by an alkali wash and heat lysis method [28]. Briefly, blood culture suspensions (0.5 mL) were mixed with alkali wash solution (0.5 M NaOH, 0.05 M sodium citrate, 1.0 mL) and then further mixed using a rotary wheel for 10 min at room temperature. The mixture was subsequently centrifuged at 13,000×g for 5 min, the cell pellet was washed twice in Tris-HCl (0.5 M, pH 8.0, 0.5 mL) and centrifuged as described above, and the resulting pellet was resuspended in Tris-EDTA (10 mM Tris-HCl, pH 8.0, 1 mM EDTA, 0.1 mL). The suspension was subsequently heated at 100 °C for 1 h. After two cycles of 5 min of freezing and 2 min of boiling, the sample was centrifuged at 13,000×g for 15 min and the supernatant was transferred to a clean tube and stored at 20 °C until use.

PCR amplification was performed with a Thermal Cycler 2400 (Applied Biosystems, Foster City, CA, USA). The PCR reaction mixture (50 μL) contained genomic DNA extract (2 μL), MgCl2 (0.3 mM), 0.01 mM of each dNTP, 0.16 μM of each primer, Taq DNA polymerase (0.1 U), 10× PCR Buffer (5 μL) and distilled water (33 μL). Amplification was performed with the following cycling conditions: 94 °C for 5 min; 25 cycles of 94 °C for 30 s, 53 °C for 45 s, 72 °C for 45 s; and a final extension at 72 °C for 10 min. The PCR products were visualized by 2.0% agarose gel electrophoresis with ethidium bromide, and then analyzed by a gel documentation system (Vilber Lourmat, France).

### 2.5. Immobilization of the oligonucleotide probes

**Figure 2.**
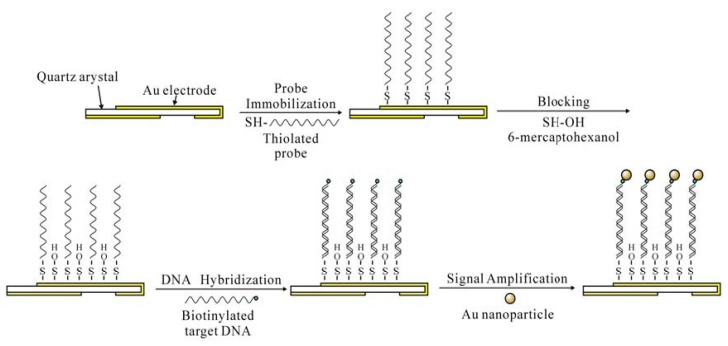
The assembly procedure of the QCM nucleic acid biosensor.

The surface chemistry of probe immobilization and detection is illustrated in Figure 2. Initially, the quartz crystals were cleaned with 1M NaOH for 20 min, 1M HCl for 5 min, and Piranha solution (30%, v/v, H_2_O2:H_2_SO_4_ = 1:3) for 1 min. Then the crystals were thoroughly washed with absolute ethanol and super pure water successively, and dried with nitrogen to obtain a clean Au surface. Subsequently, 10 detection wells were assembled with disposable crystals, and PBS (0.01 M Na_2_HPO_4_, 0.0017M KH_2_PO_4_, 0.138 M NaCl, 0.0027 M KCl, pH 7.5 at 25 °C, 90 μL) was added respectively to each detection well. The resonance frequency of the crystal was recorded until a steady baseline was obtained (F_0_) in 5 min. Then, thiolated probe solutions (10 μL) with different concentrations (0.25, 0.5, 1.0, 2.0 and 4.0 μM) of PBS were injected into each detection well. Super pure water was used as a negative control. The probes were self-assembly immobilized on the gold electrode for 1 h at room temperature (25 °C), the resonance frequency dropped down and tended to become stable, and this stable frequency was recorded as F1. The whole detection procedure was monitored in real time and frequency changes was recorded and displayed by the software BIDE 2.11. The induced frequency shifts (ΔF, ΔF□=F_1_-F_0_) were calculated automatically, and the results were given out by the software BIDE 2.11. After 3 times washing with PBS to remove unbound probes, an aqueous solution of 1 mM 6-mercaptohexanol was added into each well and left for 30 min at room temperature (25 °C) to block any non-specific interaction site.

### 2.6. Detection procedure

The positive and negative control oligonucleotides were used as target DNA for the optimization of the experimental conditions following the procedure illustrated in Figure 2. The system temperature was then adjusted to 42 °C and 90 μL PBS buffer was injected into each detection well. Then, target DNA solution (2×10^-9^ M, 10 μL) was injected into each detection well and allowed to stand for 30 min for the hybridization with the probes. The frequency shifts (ΔF) induced by hybridization were also recorded. The crystals were then washed using PBS buffer to remove the nonspecifically adsorbed nucleotides. Subsequently, the system temperature was adjusted to 37 °C and PBS buffer (90 μL) was injected into each detection well. After equilibrium for about 5 min, nanoparticle solutions (10 μL) with different concentrations (0, 0.2, 0.4, 0.6, 0.8, 1, 1.2, 1.4 mg/mL) were added, respectively. The resonance frequency was recorded as time went on. The buffer pH effects (4.0, 4.5, 5.0, 5.5, 6.0, 6.5, 7.0, 7.5, 8.0, 8.5, 9.0, 9.5, 1.0, 10.5 and 11.0) during the Au nanoparticle signal amplification were also evaluated. After the PCR amplification products were denaturized at 95 °C for 5 min followed by freezing the sample in ice for 30 s, denatured DNA solution (10 μL) was used following the same procedure used for synthetic oligonucleotides. The negative control (PCR products of 5.8×10^7^ CFU/mL *P. aeruginosa* ATCC 27853) and the blank control (PCR products using super-pure water as the PCR template) were used in the tests.

### 2.7. Electrochemical characterization of the QCM electrodes

To demonstrate the alteration of the interfacial properties of the QCM electrodes, Faradaic impedance spectra measurements were carried out with an Autolab PGSTAT30 potentiostat/impedance frequency analyzer (Eco Chemie). Electrochemical measurements were performed in a three-electrode cell consisting of the Au electrode, a glass carbon auxiliary electrode isolated by a glass frit, and saturated calomel electrode (SCE). All impedance measurements were performed in 0.17 M PBS buffer (pH 7.5), which included 0.02 M K_3_[Fe(CN)_6_]: K_4_[Fe(CN)_6_], in a 1:1 ratio, as a redox probe. An alternating voltage (10 mV) was applied at a bias potential of 0.17 V in the frequency range 0.5 Hz-20,000 Hz.

### 2.8. Clinical sample detection

To demonstrate the use of the piezoelectric nucleic acid biosensors for the identification of *S. epidermidis*, 35 positive blood culture samples and 20 negative blood culture samples were detected individually using the QCM nucleic acid array and the conventional microbiological techniques.

### 2.9. Data analysis

Each experiment was repeated five times to test the reproducibility of the QCM biosensor. All data were presented as the mean ± standard deviation (SD). SPSS 10.0 software was used for a two-tailed Student's *t*-test, and the statistical significance of *P*-values was set at the 0.05 level.

## 3. Results and Discussion

### 3.1. Immobilization of synthesized oligonucleotide probes

**Figure 3.**
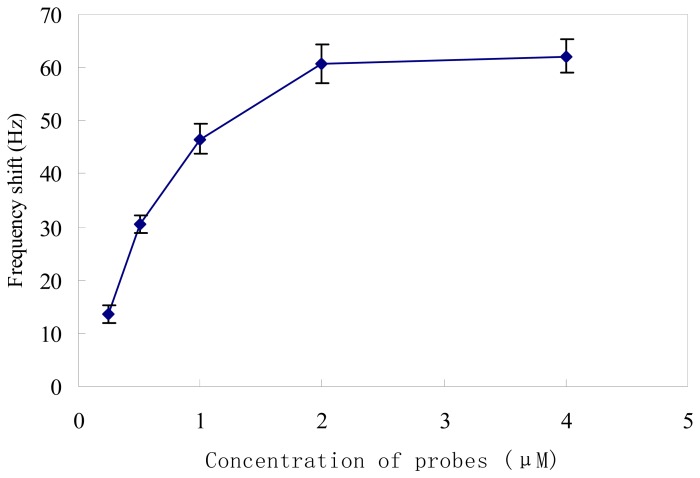
Probe immobilization efficiency in the QCM nucleic acid biosensor. Error bars indicate the standard deviation (n=5).

Methods to immobilize oligonucleotide probes on bare gold biosensor surfaces have been investigated for years, such as avidin and biotin interactions [29], Langmuir-Blodgett method [30], and the self-assembled monolayers [17, 31]. In this study, the self-assembled monolayers method was adopted because it can create reproducible, well-ordered and stable immobilization layers [31, 32]. To fabricate nucleic acid biosensors, surface density of immobilized probes is an important factor for accurate detection because a low probe surface density leads to a lower upper limit of detection, whereas redundant probes will significantly increases steric hindrance on hybridization [17, 32]. Therefore, various concentrations (0.25, 0.5, 1.0, 2.0 and 4.0 μM) of thiolated probes were used to evaluate immobilization efficiency on the gold surface of the quartz crystals. As shown in Figure 3, the ΔF increased with the increase of probe at concentrations of 0.25 to 2.0 μM. Then ΔF reached a plateau and varied little, although the probe concentration continued to increase. Probes at concentrations of 2.0 and 4.0 μM yielded the greatest covalent attachment to the gold surface compared with other concentrations (*P*<0.05). There was no significant difference between ΔF at concentrations of 2.0 and 4.0 μM (*P* > 0.05), indicating saturation of the immobilization sites on the gold surface of the quartz crystal. Considering immobilization efficiency, detection range, and detection cost, we selected the probe concentration of 2.0 μM as the optimal immobilization concentrations for the following experiments.

Although the unoccupied residues of streptavidin-coated Au nanoparticles have been blocked by the manufacturer, it was found in our study that a few streptavidin-coated Au nanoparticles could be adsorbed onto the gold surface of the electrode to cause false-positive signal. Thus, 1 mM 6-mercaptohexanol was used to block any non-specific interaction site of the electrode. The quartz crystals were modified with thiolated probes (2.0 μM), blocked using 6-mercaptohexanol, and then treated with Au nanoparticle solutions (0.6 mg/mL). There was a significant frequency shift (52±4 Hz) for the crystal without 6-mercaptohexanol blocking, while much less frequency change (<5 Hz) was found on the 6-mercaptohexanol blocked crystal. The results indicate that 6-mercaptohexanol is able to effectively prevent the non-specific adsorption of streptavidin-coated nanoparticles to the gold surface of the electrode.

### 3.2. Optimization of the experimental conditions for the signal amplifying biosensing system

**Figure 4.**
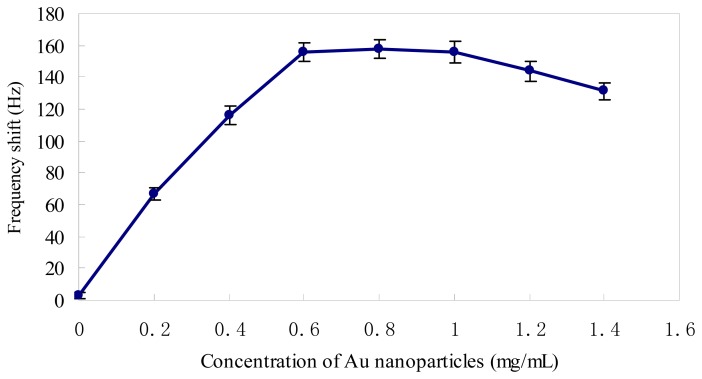
Effect of the Au nanoparticle concentrations on the frequency shifts during signal amplification procedure, frequency shift under the conditions: 2×10^-9^ M positive control and pH 7.5 at 37 °C. Error bars indicate the standard deviation (n=5).

The streptavidin-coated Au nanoparticle concentration is an important influence factor for the frequency response of this amplified mass piezoelectric biosensor array. The suitable Au nanoparticle concentration should benefit both the mass enhancement and the biomolecular interactions between avidin on the Au nanoparticles and biotin on the target DNA. As shown in Figure 4, the ΔF of crystal gradually increased with the increase of the Au nanoparticles in the range of low concentration. When the concentration was above 0.6 mg/mL, the ΔF tended to be stabilization. While the concentration of nanoparticles was higher than 1 mg/mL, the ΔF appeared to decrease instead of increasing with the buildup of Au nanoparticles, which is possibly due to the augmentation of steric hindrance in the three-dimensional space caused by redundant nanoparticles. Therefore, 0.6 mg/mL of Au nanoparticles were used in subsequent experiments.

**Figure 5.**
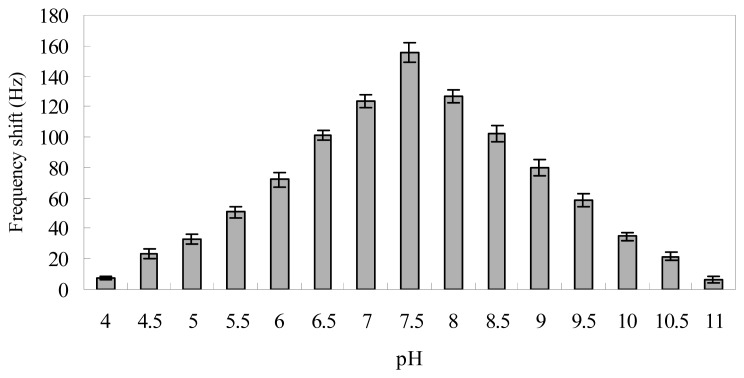
Effect of the buffer pH on the frequency shifts during signal amplification procedure, frequency shift under the conditions: 2×10^-9^ M positive control and 0.6 mg/mL Au nanoparticles at 37 °C. Error bars indicate the standard deviation (n=5).

As the buffer pH strongly affects the conjugation of Au nanoparticles to biotinylated target DNA and the stability of the complex of probe and target DNA [33-36], its effect on the signal amplification of Au nanoparticles is shown in Figure 5. The ΔF caused by the conjugation of Au nanoparticles to positive oligonucleotides (2×10^-9^ M) increased rapidly when PBS buffer pH increased from 4 to 7, and the ΔF decreased rapidly when PBS buffer pH increased from 8 to 11. The maximum frequency shift due to the conjugation was found at pH 7.5. The possible explanation may be that the formed dsDNA would be denatured in acidic or alkaline media and the re-hybridization of target and probe nucleotides would be hindered by the increase of electrostatic repulsion between anionic chains, which would cause the dsDNA layer damaged and decrease the ΔF [34, 35]. Moreover, the binding of biotinylated target DNA to streptavidin-coated Au nanoparticles is likely to be counteracted by the increase of electrostatic repulsion which would make close packing thermodynamically unfavorable, and the lower structural stability of the protein molecules away from pI, as observed also by Sun *et al.* [36]. These effects are likely cooperative in generating the observed behavior. Thus, PBS buffer pH at 7.5 was used in subsequent experiments.

### 3.3. Validation of the fabrication and detection of the QCM biosensor by electrochemical impedance spectroscopy

**Figure 6.**
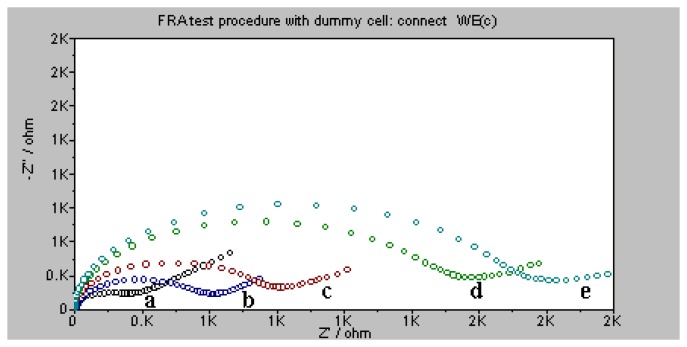
Nyquist diagram (Z_im_ vs. Z_re_ ) of electrochemical impedance measurements corresponding to the following: **(a)** the bare electrode; **(b)** 2.0 μM thiolated probe modified electrode; **(c)** 1 mM 6-mercaptohexanol modified electrode; **(d)** after the hybridization with 2×10^-9^ M positive control; **(e)** after the signal amplification with 0.6 mg/mL Au nanoparticles. All impedance measurements were performed in 0.17 M PBS buffer, pH 7.5.

When materials are adsorbed onto gold surfaces, they form a dense layer which inhibits the interfacial electron transfer. Electrochemical impedance spectroscopy is used as a technique, which can give information on the impedance changes of surface modified electrodes [37]. In this study, Faradaic impedance spectra measurements were performed to further confirm the stepwise modification of the QCM electrodes. Figure 6 shows the Faradaic impedance spectra (in the form of a Nyquist plot). The complex impedance was presented as the real (Z_re_) and imaginary (Z_im_) components that originated mainly from the resistance and capacitance of the cell, respectively. It can be seen that the electrochemical impedance spectroscopy of bare gold electrode has very low resistance (curve a). The self-assemblies of thiolated probes (2.0 μM) and 6-mercaptohexanol (1 mM) on the electrode increased the interfacial electron-transfer resistances (curve b and c), indicating the successful probe immobilization and 6-mercaptohexanol blocking. The hybridization of target DNA (positive control oligonuleotide, 2×10^-9^ M) and probes generated a transfer insulation for the redox label, resulting in an increase in electron-transfer resistance at the electrode (curve d). In the last step of Au nanoparticle signal amplification, the electron-transfer resistance became larger because that the decorated nanoparticles (0.6 mg/mL) just combined with the probes captured target DNA by the biotin-avidin bonding and resulted in a more insulating layer (curve e). Those results well demonstrate the fabrication and detection process on the quartz crystal.

### 3.4. Detection of PCR-amplified DNAs

**Figure 7.**
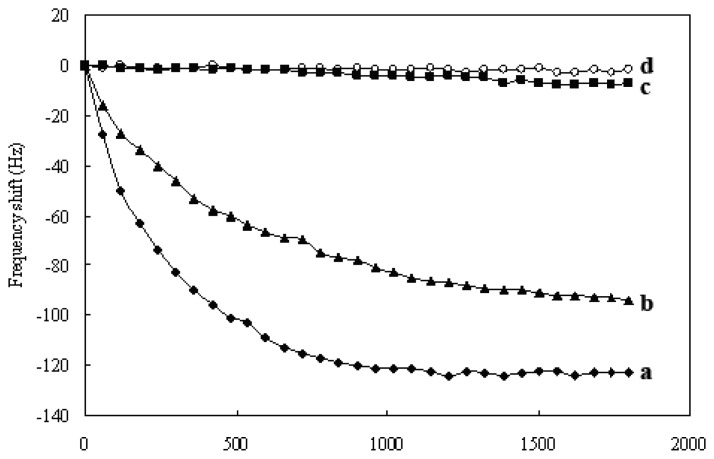
Typical characteristics of frequency shift that correspond to (a) the black line and solid diamond, the detection procedure of PCR products of 1.3×10^7^ CFU/mL *S. epidermidis* ATCC 12228 with 0.6 mg/mL Au nanoparticles amplification; (b) the black line and solid triangle, the detection procedure of PCR products of 1.3×10^7^ CFU/mL *S. epidermidis* ATCC 12228 without Au nanoparticles amplification; (c) the black line and solid squares, the detection procedure of blank control; (d) the black line and circles, the detection procedure of negative control.

Detection sensitivity of QCM nucleic acid biosensor depends on the mass change on the gold surface of crystal [17]. In this study, we added streptavidin-coated Au nanoparticles into the reaction system to amplify the signal of PCR-amplified DNA detection. Figure 7 shows the typical frequency curves of the amplified mass QCM nucleic acid biosensor. The PCR-amplified target DNA (PCR products of 1.3×10^7^ CFU/mL *S. epidermidis* ATCC 12228) was first applied to the biosensor to hybridize with the probe, resulting a frequency drop (curve b). Then, with the addition of 10 μL of Au nanoparticle solutions (0.6 mg/mL), the resonant frequency rapidly decreased and about 30 min later, tended towards stabilization (curve a). The ΔF caused by the conjugation of Au nanoparticles to target DNA (122±4 Hz) was significantly larger than that caused by hybridization with target DNA (91±5 Hz) (*P*<0.05). The streptavidin-coated Au nanoparticles enhanced the ΔF by approximately 1.3-fold while the Au nanoparticles were conjugated to probe captured target DNA. Blank and negative control tests were carried out by substituting the target PCR products with the blank control (PCR products using super-pure water as the PCR template) and the negative control (PCR products of 5.8×10^7^ CFU/mL *P. aeruginosa* ATCC 27853). Although the streptavidin-coated Au nanoparticles were also applied to the blank and negative controls tests, the Au nanoparticles failed to induce significant frequency shifts (curve c and d), showing that non-specific adsorption of streptavidin-coated Au nanoparticles did not result in an obvious frequency shift. The results indicate that the binding of biotinylated target DNA to streptavidin-coated Au nanoparticles is a specific interaction, and represents the amplification of signal. In addition, the Faradaic impedance spectra corresponding to the similar experiments were performed under the same conditions. The electron-transfer resistance (R_et_) increased from 1325 Ω to 1712 Ω upon the formation of the assembly between the biotinylated target DNA and streptavidin-coated Au nanoparticles, whereas in the blank and negative controls tests the streptavidin-coated Au nanoparticles only resulted in a minute change in the interfacial electron-transfer resistance (ΔR_et_<51 Ω). Thus, these results show that the signal amplification is attributed to the specific Au nanoparticles to target DNA assembly.

**Figure 8.**
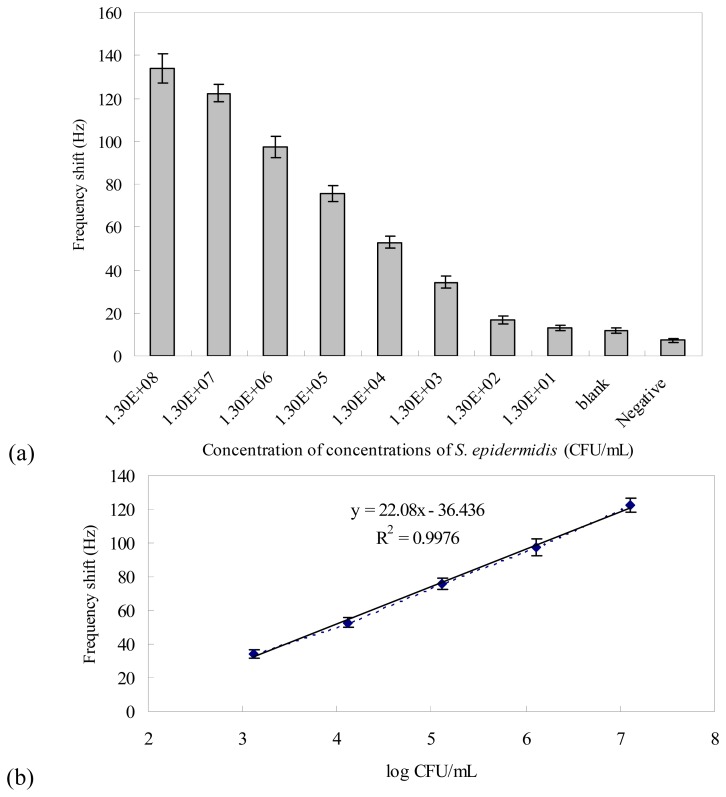
(a) The responses of the QCM sensor to the PCR-amplified DNAs isolated from different concentrations of *S. epidermidis*; (b) A linear relationship between the frequency shift vs. log (CFU/mL of *S. epidermidis*) was found from 1.3×10^3^ to 1.3×10^7^ CFU/mL. Error bars indicate the standard deviation (n=5).

Moreover, for quantitative detection of *S. epidermidis*, genomic DNA was extracted from different concentrations of serially diluted *S. epidermidis* ATCC 12228 (1.3×10^1^ to 1.3×10^8^ CFU/mL) using the protocol described before. PCR was carried out using the DNA extracted from each concentration of cells. The PCR products were detected using gel electrophoresis to confirm the successful amplification of PCR products with the right size, 319-bp (data not shown). The PCR-amplified DNAs for *S. epidermidis* (1.3×10^1^ to 1.3×10^8^ CFU/mL) were detected by the QCM nucleic acid biosensors using Au nanoparticles as amplifiers under the optimal experimental conditions. The frequency shifts resulted from the detections are shown in Figure 8a. The ΔF of the sensor was enhanced as the cell concentration of the PCR products increased. The blank control (PCR products using super-pure water as the PCR template) and the negative control (PCR products of 5.8×10^7^ CFU/mL *P. aeruginosa* ATCC 27853) were also detected. The measurements were highly reproducible for all concentrations of *S. epidermidis* (n=5, RSD<11.8%). The detection limit was taken when the signal is 3-fold more than the noise. The frequency change of the 1.3×10^3^ CFU/mL was three times bigger than it, so the detection limit of our system was assessed as 1.3×10^3^ CFU/mL. The detection limit of the Au amplification QCM nucleic acid biosensor is one order lower than that of our non-amplification QCM nucleic acid biosensor test (1.3×10^4^ CFU/mL) as well as the result (1.2×10^4^ CFU/mL) of previously reported QCM biosensor without signal amplifiers [23]. We expect this analytical sensitivity of our QCM system to be sufficient for detecting *S. epidermidis* from positive blood cultures. A linear relationship was found between the frequency shift vs. logarithmic number of cell concentration from 1.3×10^3^ to 1.3×10^7^ CFU/mL (y=22.08x-36.436, R^2^=0.9976) (Figure 8b). The result shows that the *S. epidermidis* cell concentration was positively relative to the response of the frequency shift indicating that it is possible to enumerate *S. epidermidis* using the QCM nucleic acid biosensor array. However, higher cell concentrations (1.3×10^7^ to 1.3×10^8^ CFU/mL) yielded similar frequency shifts and the detection signals were not distinguishable from each other (*P* > 0.05), which was possibly because the PCR reaction had reached the plateau stage [22].

### 3.5. Specificity of the QCM system in detecting S. epidermidis

**Figure 9.**
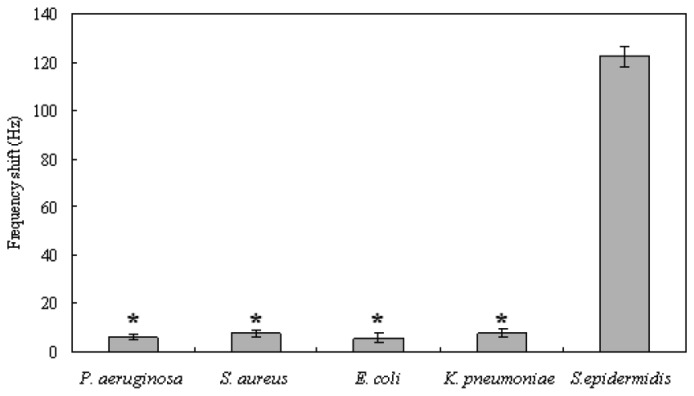
Frequency shifts of the QCM biosensor for PCR-amplified DNAs of 5 bacteria. PCR products of *S. epidermidis* (ATCC 12228), *P. aeruginosa* (ATCC 27853), *S. aureus* (ATCC 25923), *E. coli* (ATCC 25922), and *K. pneumoniae* (ATCC 700603) with the concentration of 1.3×10^7^ CFU/mL were detected by the Au nanoparticle signal amplification based QCM biosensors. Error bars indicate the standard deviation (n=5). * indicates *P* < 0.05 vs. PCR products of 1.3×10^7^ CFU/mL *S. epidermidis* ATCC 12228.

In addition, we wanted to determine the specificity of our system. To do this, genomic DNA was extracted from *S. epidermidis* (ATCC 12228), *P. aeruginosa* (ATCC 27853), *S. aureus* (ATCC 25923), *E. coli* (ATCC 25922), and *K. pneumoniae* (ATCC 700603) with the concentration of 1.3×10^7^ CFU/mL. PCR amplification and QCM nucleic acid biosensor detections were carried out using the same protocol as described before. Although the 16S rRNA gene presents in these bacterial strains, the probe which was designed for specific hybridization with *S. epidermidis* 16S rRNA gene fragment could not successfully capture the 16S rRNA gene fragments from *P. aeruginosa*, *S. aureus*, *E. coli*, and *K. pneumonia*. As shown in Figure 9, we compared the frequency shifts for the detections of five bacteria. The results show that non-specific PCR-amplified DNAs fail to result in significant frequency shifts (*P* < 0.05), indicating that this QCM system has relatively low cross-reactivity and acceptably high specificity. These results suggest that the QCM system established in this study may be specifically used in *S. epidermidis* detection.

#### 3.6. Clinical sample detection and comparison

Furthermore, to demonstrate the use of the QCM nucleic acid biosensor for the determination of *S. epidermidis* in clinical samples, 35 positive blood culture samples and 20 negative blood culture samples were tested. All the samples have been previously detected by the reference method using the conventional microbiological techniques. In this test, 34 of 35 case samples were found to have a detectable frequency change, suggesting that these samples are *S. epidermidis* positive. In one sample the additionally performed PCR was negative, which have not been correctly identified, presumably due to failure of the nucleic acid extraction or the presence of inhibitors in the extracted DNA [38]. Compared with conventional microbiological method, the diagnostic sensitivity of the assays resulted in 97.14 % (the success rate in distinguishing cases within the pool of case samples), while the specificity resulted in 100 % (the success rate in distinguishing controls within the pool of control samples) (Table 2).

This test system allows identification of *S. epidermidis* within ca. 4 to 5 h, including DNA isolation, PCR amplification, and detection by QCM nucleic acid biosensor array. Therefore, in contrast to conventional culture and biochemical identification techniques, which usually take 1 to 2 days, the QCM system generates results much quicker. The costs of the kit and reagents are moderate and amount to ca. $3.50 per sample and test. These results suggest that it is feasible to apply the proposed Au nanoparticle signal amplification based QCM nucleic acid biosensor array to detect *S. epidermidis* in clinical diagnostics.

### 4. Conclusions

In this paper, a sensitive QCM nucleic acid biosensor array based on Au nanoparticle signal amplification for the detection of *S. epidermidis* was developed and demonstrated. The use of Au nanoparticles effectively amplified the signals in frequency shifts due to their relatively large mass compared to DNA targets, and resulted in a improved detection limit for *S. epidermidis* (1.3×10^3^ CFU/mL for PCR products) without any enrichment of the culture. In addition, a quantitative relationship was noted between the measured signal and the concentration of *S. epidermidis* cells in range of 1.3×10^3^ to 1.3×10^7^ CFU/mL. Moreover, for clinical blood sample detection, the clinical sensitivity is 97.14% and the clinical specificity is 100% compared with the conventional microbiological method. The detection of *S. epidermidis* could be completed within ca. 4 to 5 h. Our results suggest that this QCM nucleic acid biosensor array with streptavidin-coated Au nanoparticles as amplifiers can be expected to be a potential clinic diagnosis method for rapid detection of *S. epidermidis* or other microorganisms in clinical samples.

## Figures and Tables

**Table 1.** Sequences of oligonucleotides used in this study.

**Oligonucleotide**	**Sequence**
Thiolated probe	5′-HS-(CH_2_)_6_-CGAGCGAACAGATGAGGAGC-3′
Forward primer	5′-TGGCGGCGTGCCTAATACATG-3′
Reverse primer	5′-biotin-CCCGTAGGAGTCTGGACCGTGTC-3′
Positive control oligo	5′-biotin-GCTCCTCATCTGTTCGCTCG-3′
Negative control oligo	5′-biotin-CGAGCGAACAGATGAGGAGC-3′

**Table 2.** Comparison of conventional microbiological method and QCM biosensor for detection of *S. epidermidis*.

**Method**	**No. of blood cultures**		

	**No. positive**	**No. negative**	**n**
conventional microbiological method	34	21	55
QCM biosensor	35	20	55
